# Synthesis of an Environmentally Friendly Modified Mulberry Branch-Derived Biochar Composite: High Degradation Efficiency of BPA and Mitigation of Toxicity in Silkworm Larvae

**DOI:** 10.3390/ijms24043609

**Published:** 2023-02-10

**Authors:** Han Qu, Lin Chen, Fujian Yang, Jiangwei Zhu, Chengdu Qi, Guilong Peng

**Affiliations:** 1 State Key Laboratory of Silkworm Genome Biology, Key Laboratory of Sericultural Biology and Genetic Breeding, Ministry of Agriculture and Rural Affairs, College of Sericulture, Textile and Biomass Sciences, Southwest University, Chongqing 400715, China; 2 Key Laboratory of Eco-Environment of Three Gorges Region of Ministry of Education, Chongqing University, Chongqing 400045, China; 3 Co-Innovation Center for Sustainable Forestry in Southern China, Nanjing Forestry University, Nanjing 210037, China; 4 School of Environment, Nanjing Normal University, Nanjing 210023, China

**Keywords:** biochar, bisphenol A, persulfate, silkworm, toxicity

## Abstract

In the present study, mulberry branch-derived biochar CuO (MBC/CuO) composite was successfully synthesized and used as a catalyst to activate persulfate (PS) for the degradation of bisphenol A (BPA). The MBC/CuO/PS system exhibited a high degradation efficiency (93%) of BPA, under the conditions of 0.1 g/L MBC/CuO, 1.0 mM PS, 10 mg/L BPA. Free radical quenching and electron spin-resonance spectroscopy (ESR) experiments confirmed that both free radicals ^•^OH, SO_4_^•−^ and O_2_^•−^ and non-radicals ^1^O_2_ were involved in the MBC/CuO reaction system. Cl^−^ and NOM displayed negligible influence on the degradation of BPA, while HCO_3_^−^ promoted the removal of BPA. In addition, the toxicity tests of BPA, MBC/CuO and the degraded BPA solution were conducted by the 5th instar silkworm larvae. The toxicity of BPA was reduced after the treatment in the MBC/CuO/PS system, and no obvious toxicity of the synthesized MBC/CuO composite was found in the toxicity evaluation experiments. This work provides a new value-added utilization of mulberry branches as a cost-effective and environmentally friendly PS activator.

## 1. Introduction

Biochar (BC) is a type of porous carbonaceous material that can be obtained through the pyrolysis of a variety of biomass of plants or animals under anoxic conditions [1,2]. It has attracted considerable attention in the remediation of hazardous pollutants in wastewater due to its extremely low cost and environmental friendliness. The conversion of various biomasses to BC has been reported in much of the literature [3,4,5,6], including activated sludge, animal manure, agricultural wastes, or carbonaceous sources such as bamboo, sawdust, and coconut shells. Currently, a large amount of agricultural waste is discarded every year, which poses a great challenge to the safe disposal and utilization of carbon-rich biomass residues.

Mulberry (*Morus* spp.) is an important economic crop with the characteristics of fast growth and strong adaptability and is widely planted in many areas of China. However, the branches are currently primarily discarded as agricultural waste once the leaves are harvested, and traditional incineration methods not only fail to utilize resources efficiently, but also cause severe air pollution (greenhouse gas emissions), which has been strictly prohibited in China [7]. Therefore, it is a desirable alternative if mulberry branches can be converted into prospective value-added biochar using appropriate pathways, and this would greatly increase mulberry’s economic value. Converting waste mulberry branch biomass into BC and using it for water pollution remediation would be a “win–win” solution for improving waste management and protecting the environment. Although biochar has been extensively explored in recent years for utilization as a persulfate (PS) activator in degrading organic pollutants, its catalytic ability is still very limited [8,9,10]. Recently, BC has been widely used as a support for catalysts to activate PS degradation of organic pollutants, which have been proven to synergistically enhance catalytic performance [11]. Typically, biochar acts as an ideal carrier for dispersing and stabilizing nanoparticles to enhance their catalytic ability, and modified biochar can enhance its catalytic activation performance compared to when pristine [12]. Metal oxides such as Co, Fe, Mn, and Cu are considered efficient catalysts for PS activation, especially CuO-based catalysts, as efficient, economical, and low-toxicity catalysts, have attracted extensive attention for the removal of pollutants by activating PS [13]. However, during the oxidative degradation processes, based on the metal oxide catalysis system, more toxic intermediates may be produced, which is harmful to the ecological environment and even human beings. Therefore, it is of great importance to evaluate the toxicity of the final reaction solution during the treatment process and develop highly efficient and environmentally friendly materials to solve the environmental pollution problem [14]. Silkworm (*Bombyx mori*; *Lepidoptera: Bombycidae*) is an ideal invertebrate model in life sciences, with a short growth cycle and fast reproduction. Silkworms have similar pharmacokinetics and deliver a half-lethal dose to mammals, such as rabbits, rats, mice, and so on [15]. Additionally, silkworms are usually used as an animal model due to their low stress, high disease resistance, sensitivity to poisons, and ability to detect most of the harmful environmental substances in related low levels [15,16,17].

In this work, mulberry branch-derived biochar (MBC) was prepared and used as a support for CuO. Subsequently, a MBC/CuO composite was synthesized using the chemical coprecipitation method. Then, the typical environmental endocrine disrupting hormone bisphenol A (BPA) [18], which is frequently detected in the aquatic environment, was selected to evaluate the catalytic performance of MBC/CuO towards PS. Various parameters affecting the degradation efficiency of BPA, including the dosage of MBC/CuO and PS, pH, inorganic ions, and natural organic matter (NOM) were investigated. Free radical quenching and electron spin-resonance spectroscopy (ESR) experiments were performed to prove the presence radical species in the reaction system. Furthermore, in order to provide more information on the overall environmental risk, the toxicity of the final degradation solution and prepared MBC/CuO was estimated using silkworm larvae.

## 2. Results and Discussion

### 2.1. Characterization of the Samples

**Figure 1:**
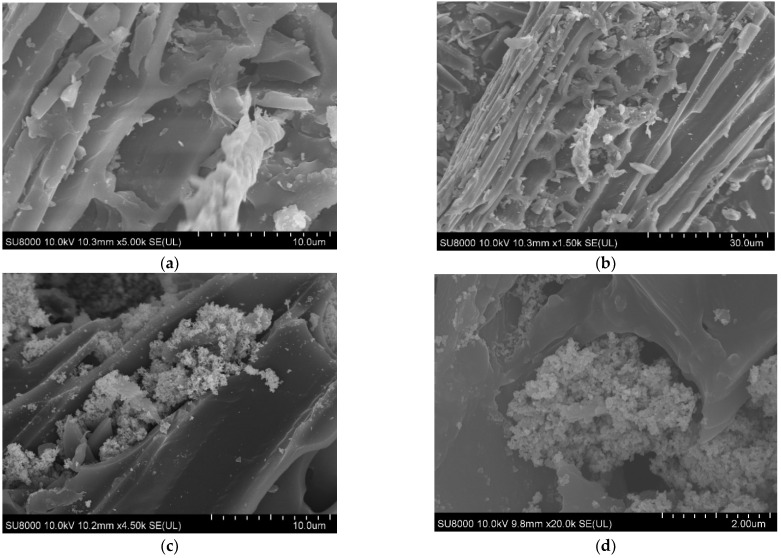
SEM images of MBC (**a**,**b**) and MBC/CuO (**c**,**d**).

The morphology of the MBC and MBC/CuO was observed using SEM and the images are shown in Figure 1. The surface of the MBC was porous, with narrow tubular and well-dispersed channels (Figure 1a,b), and form hollow structural carbon with interconnected macropores with the size of 1–6 µm. In Figure 1c,d, some snowflake-like CuO particles were attached to the inside and outside of the hollow tube walls of MBC. The porous structure of MBC/CuO could provide more exposed active sites for PS activation and organic pollutant degradation. Meanwhile, the specific surface areas and pore characteristics of the MBC and MBC/CuO are shown in Table S2. After loading CuO nanoparticles, the BET surface area of MBC decreased from 84.9 m^2^/g to 82.9 m^2^/g (Table S2). This decrease can be attributed to the partial blockage of MBC pores by CuO nanoparticles. As shown in Figure S2a,b, the cumulative pore volume greatly decreased after the load of CuO onto MBC. Noteworthy, the pore of MBC/CuO in the size of 1–2 nm increased, while in 2–8 nm decreased, compared to MBC, further indicating that the preparation process of MBC/CuO blocked the mesopore and extruded the micropore. The XRD patterns of the as-synthesized MBC, MBC/CuO, and CuO are depicted in Figure S2c. XRD analysis showed that MBC was amorphous carbon, and a wide peak at 2θ of 20–25° was attributed to the carbon characteristic diffraction peak. The characteristic peaks at 32.35°, 35.66°, 38.73°, 48.72°, 53.33°, 58.19°, 61.53°, 66.13°, 68.18°, and 75.09° are corresponding to (110), (−111), (111), (−202), (020), (202), (−113), (−311), (−220), and (−222) planes of CuO [13,19], respectively. The XRD pattern of MBC/CuO completely matches the characteristic peaks of MBC and CuO, indicating that the MBC/CuO composites were successfully synthesized.

### 2.2. Degradation Efficiency of BPA in Different Systems

**Figure 2:**
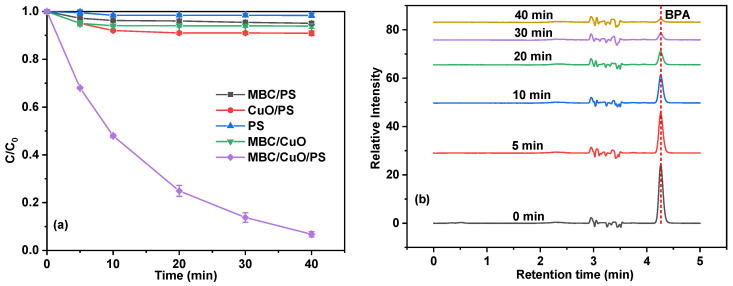
BPA removal in different systems (**a**) and the change of HPLC peaks of BPA in the reaction solution at different reaction times (**b**). Experimental conditions: (BPA) = 10 mg/L, (PS) = 1 mM, (catalyst) = 0.1 g/L, the mass ratio of CuO to MBC for the used MBC/CuO was 1:1, pH = 3.84, T = 25 °C.

The degradation kinetics of BPA were evaluated in different reaction systems along with time. As shown in Figure 2a, although PS is considered a strong oxidant, no obvious removal effect on BPA was observed in PS alone group, indicating that PS was not effective enough to oxidize BPA within 40 min [20]. It has been reported that BC can be used as adsorbent to remove BPA [21]. However, in the present study, the removal of BPA in the MBC/CuO system was only 6%, which means that adsorption ability of BC for BPA was very limited. On the other hand, although CuO and MBC can activate PS to degrade organic pollutants, which has been reported in much of the literature [22,23,24], the removal of BPA in MBC/PS and CuO/PS systems were only 5% and 9%, respectively. On the one hand, the catalytic activation performance of MBC was very limited. On the other hand, CuO particles tend to agglomerate together, resulting in the unsatisfactory catalytic performance. In contrast, the MBC/CuO/PS system exhibited much higher removal efficiency and up to 93% of BPA was removed. As seen in Figure 2b, in the MBC/CuO/PS system, the removal of BPA was very obvious, the peak intensity of BPA at the retention time of 4.25 min declined rapidly with the extension of reaction time. The kinetics of BPA degradation was well-fitted with the pseudo-first-order kinetics model and the rate constants (*k_obs_*) of BPA in the MBC/CuO/PS systems was 0.066 min^−1^ (Figure S3). The high activation ability of MBC/CuO might be attributed to the surface −OH groups bonded with CuO can accelerate PS activation and generate SO_4_^•−^ through the rapid electron transfer between MBC/CuO and PS [20,24,25]. Furthermore, the introduction of MBC greatly enhanced the dispersion of CuO, which was conducive to the activation of PS. In order to further demonstrate the superior catalytic performance of MBC/CuO, the removal of BPA in the MBC/CuO/PS system was also compared with previous research results. As indicated in Table S3, compared with these selected catalysts, the *k_obs_* obtained in the present work was the largest (0.066 min^−1^) and the reaction time was the shortest (40 min), it could be concluded that MBC/CuO is an excellent catalyst for PS activation.

### 2.3. Effect of the Main Parameters on the Degradation of BPA

#### 2.3.1. Effect of the Mass Ratio of CuO to MBC

**Figure 3:**
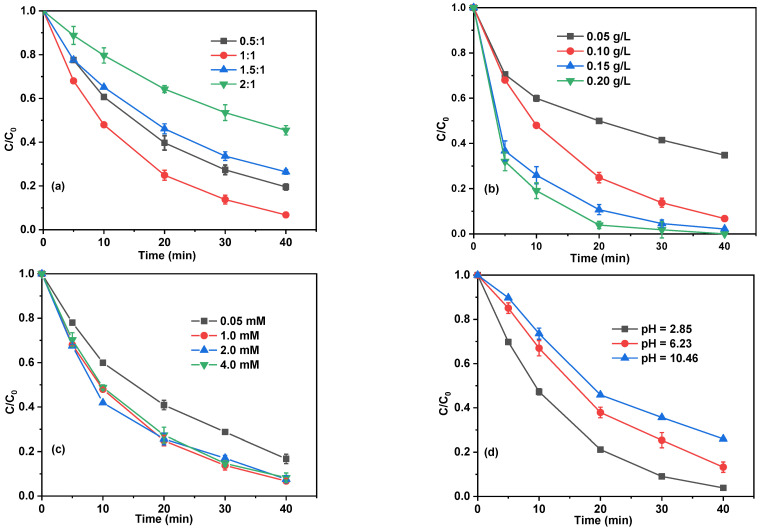
Effects of the mass ratio of CuO to MBC (**a**), catalyst dosage (**b**), PS dosage (**c**), and pH (**d**) on BPA degradation efficiency. Experimental conditions: (BPA) = 10 mg/L, (PS) = 1 mM, (catalyst) = 0.1 g/L, pH = 3.84, T = 25 °C (except for the investigated parameters).

The influence of the mass ratio of CuO to MBC in the MBC/CuO composites on the degradation of BPA was explored, and the results were displayed in Figure 3a. In Figure 3a, the removal of BPA was 80%, 93%, 74%, and 55% when the mass ratio of CuO to MBC was 0.5:1, 1:1, 1.5:1, and 2:1, respectively, suggesting that a higher CuO to MBC mass ratio (>1:1) disfavored the degradation of BPA. The reason leading to these results can be explained by the fact that when the mass ratio of CuO to MBC exceeded 1:1, a large number of CuO particles could not be uniformly dispersed on the surface of MBC, most of the CuO particles were agglomerated together, and the active sites could not be fully utilized.

#### 2.3.2. Effect of MBC/CuO and PS Dosage

In the present study, MBC/CuO and PS are two essential constituents of the MBC/CuO/PS system. The dosage of MBC/CuO on influence of the degradation efficiency of BPA was investigated in the range of 0.05–0.2 g/L. As found in Figure 3b, the degradation efficiency of BPA was 65%, 93%, 97% by using 0.05, 0.1, and 0.15 g/L of MBC/CuO, respectively, and the degradation efficiency could reach approximately 100% in 30 min with 0.2 g/L of MBC/CuO. The *k_obs_* of BPA degradation in the MBC/CuO/PS system increased from 0.0238 to 0.1324 min^−1^ when the investigated MBC/CuO dosage increased from 0.05 to 0.2 g/L. This is because more MBC/CuO provides more catalytic active sites for PS activation, leading to more reactive species production and more fast degradation of BPA [20,26].

Furthermore, the effect of PS dosage on the degradation efficiency of BPA was also evaluated in the range of 0.5 to 4.0 mM. As depicted in Figure 3c, the degradation efficiencies of BPA in the MBC/CuO/PS system with the PS dosage of 0.5, 1.0, 2.0, and 4.0 mM were 83%, 93%, 92%, and 91%, respectively. These results may imply that as the initial PS dosage increased from 0.5 to 1.0 mM, more reactive species could generate, resulting in a higher degradation degree of BPA. However, when the PS dosage further increased from 1.0 to 4.0 mM, the degradation efficiencies of BPA were almost unchanged, and even slightly inhibit the removal of BPA, suggesting 1.0 mM can be used as the optimal dosage of PS in the MBC/CuO/PS system to achieve the highest BPA degradation efficiency. Although more PS could produce a higher amount of reactive species, the excess SO_4_^•−^ could recombination using Equation (1) [27], leading to the inhibitory effect.

SO_4_^•−^ + SO_4_^•−^ → S_2_O_8_^2−^ (1)

#### 2.3.3. Effect of Initial pH

The pH value of solution is one of the most important factors affecting the catalytic degradation of organic pollutants in the PS activation systems due to its role in the surface charge property of the catalyst and the dissociation of PS and pollutants [22,28,29]. It must be noted that adding PS to the unbuffered aqueous solution causes a significant decrease in the pH value of the solution; thus, the initial solution pH was adjusted to the desired values after adding PS. Thus, degradation experiments at pH of 2.85, 6.23 and 10.46 were performed. As found in Figure 3d, the degradation efficiency of BPA was 94%, 87%, and 74%, with initial pH of 2.85, 6.23, and 10.46, respectively. The *k_obs_* values of BPA degradation calculated were 0.0817 (R^2^ = 0.994), 0.0506 (R^2^ = 0.9941), and 0.035 min^−1^ (R^2^ = 0.9904) for pH 2.85, 6.23, and 10.46, respectively, indicating that acidic condition was more favorable for the removal of BPA in the MBC/CuO/PS system in this study. The explanation for this could be attributed to the fact that the breakdown of persulfate into sulfate free radicals occurs easily under acidic conditions when using Equations (2) and (3) [30]. In addition, a relatively higher concentration of Cu^2+^ was leached at acid conditions (see Table S4), which could enhance the activation of PS. Moreover, the zero point of charge (pH_zpc_) of MBC/CuO was measured to be about 6.5, and when the pH was higher than this value, the surface of the MBC/CuO was expected to be negatively charged, and the electron repulsion between MBC/CuO and negatively charged S_2_O_8_^2−^ also inhibited the catalytic process at pH > 6.5. The hydroxyl groups on the surface of MBC/CuO are also very important to facilitating redox catalysis [31]. At lower pH, the surface −OH groups were disrupted due to the protonation of the ^−^OH on the surface of the MBC/CuO, and electrostatic attraction occurred between the negatively charged PS and the catalyst, which promoted the activation process [32].

The initial and final pH values of the reaction solutions are similar (Table S5). It is observed that the final solution pH after 40 min reaction with an initial pH of 6.23 and 10.46 decreased to 5.48 and 6.53, respectively. The decrease in the final pH after the reaction might be attributed to the generation of H^+^ after PS activation [22] and the formation of acidic small molecular intermediates in the degradation reaction [33]. On the contrary, as for the initial pH of 2.85, the final pH value increased, which might be ascribed to the reaction of CuO with H^+^.

S_2_O_8_^2−^ + H^+^ → HS_2_O_8_^−^ (2)

HS_2_O_8_^−^ → SO_4_^•−^ + SO_4_^2−^ + H^+^ (3)

#### 2.3.4. Influence of Inorganic Ions and Natural Organic Matter

**Figure 4:**
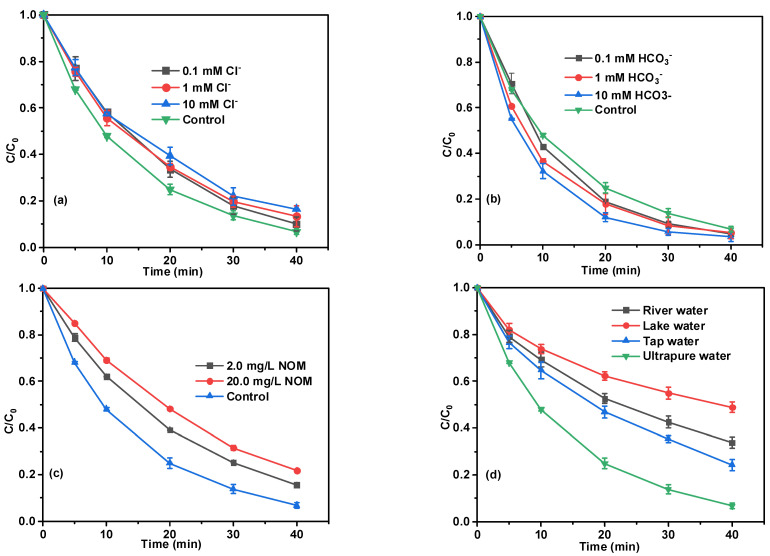
Effects of the Cl^−^ (**a**), HCO_3_^−^ (**b**), NOM (**c**), as well as water type (**d**) on BPA degradation efficiency. Experimental conditions: (BPA) = 10 mg/L, (PS) = 1 mM, (catalyst) = 0.1 g/L, pH = 3.84, T = 25 °C.

Along with reaction parameters, inorganic anions (Cl^−^, HCO_3_^−^) and NOM are widely existent in natural water and wastewater, which may interact with target pollutants and reactive species, having an significant impact on the reaction system [34]. As depicted in Figure 4a, the final BPA degradation efficiency gradually decreased from 93% to 90%, 87%, and 84% in the presence of 0.1, 1.0, and 10.0 mM Cl^−^, respectively. The suppression effect of Cl^−^ was due to the fact that chloride ions with ^•^OH and SO_4_^•−^ and form radical species with lower redox potentials (Cl^•^, Cl_2_^•−^, and ClOH^•−^) using Equations (4)–(7) [35,36,37,38], thus decreasing the total degradation efficiency of BPA. Although Cl^−^ showed a certain inhibitory effect, its degradation efficiency was still considerable. Figure 4b displayed the influence of HCO_3_^−^ on the removal of BPA. Interestingly, we discovered that the presence of HCO_3_^−^ had no significant negative effect on the removal of BPA, which was different from most of the previous reports [34,39,40]. In contrast, the degradation of BPA showed a weak promoting effect in the presence of HCO_3_^−^, this probable reason may be explained by the fact that PS can react with HCO_3_^−^ to generation HCO_4_^−^, and the generated HCO_4_^−^ could react with phenolic compounds directly though the single-electron abstract mechanism [41]. As shown in Figure 4c, the presence of various concentrations of NOM has a significant inhibitory effect on the removal of BPA. The degradation efficiency of BPA decreased to 87% and 78% within 40 min with increasing NOM concentration from 2.0 to 20.0 mg/L, respectively, indicating the inhibitory effect became more obvious with the increase of NOM concentration. This result could be reasonably explained by the fact that NOMs can act as radical scavengers due to the carboxyl groups and phenolic hydroxyls [42]. The negative effects of NOM on the degradation of organic pollutants by activated PS was also reported in other studies [43,44]. The catalytic performance of MBC/CuO/PS system for the removal of BPA in actual waters (river water, lake water, and tap water) was also explored and compared with that of ultrapure water. As depicted in Figure 4d, the removal efficiency of BPA in the river water, lake water, and tap water was only 66%, 51%, and 76%, respectively, which were much lower than that in ultrapure water (93%). The obvious inhibition in TW and RW can be explained by the presence of relatively high TOC and Cl^−^ in actual water (Table S6), which could consume free radicals [45,46].

SO_4_^•−^ + Cl^−^ → SO_4_^2−^ + Cl^•^ (4)

Cl^•^ + Cl^−^ → Cl_2_^•−^ (5)

^•^OH + Cl^−^ → ClOH^•−^ (6)

ClOH^•^− + H^+^ → Cl^•^ + H_2_O (7)

### 2.4. Activation Mechanisms

**Figure 5:**
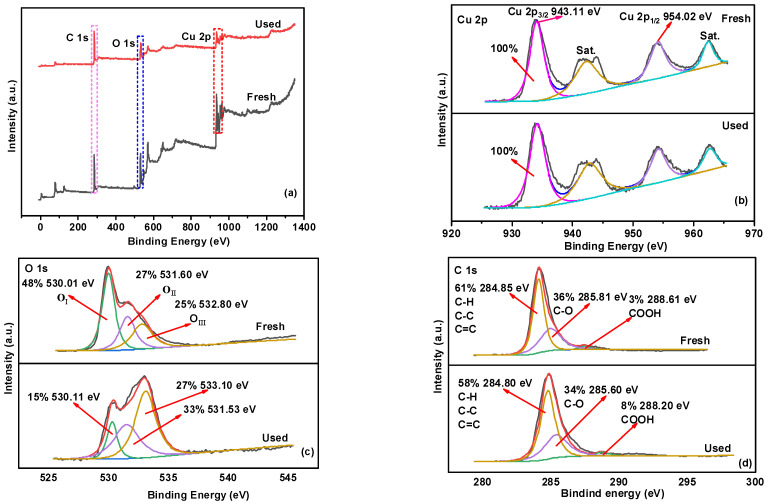
XPS spectra of survey (**a**) C 1s; (**b**) Cu 2p; (**c**) O 1s; (**d**) C 1s; on MBC/CuO before and after the reaction.

The element compositions and binding states of the fresh and used MBC/CuO were analyzed using XPS. The survey spectra indicated that only C, O, and Cu on the surface of fresh and used MBC/CuO and no obvious change in the peaks intensities of these elements could be observed between the fresh and the used MBC/CuO (Figure 5a), indicating that the catalyst has good stability. High-resolution XPS of Cu 2p were displayed in Figure 5b, the peaks centered at 943.1 eV and 954.0 eV could be attributed to the Cu 2p_3/2_ and Cu 2p_1/2_, and the two satellite peaks located at about 942.41 and 962.64 eV could be seen, which were the characteristic signal peaks of Cu^2+^, indicating the presence of CuO in the catalyst. After the degradation reaction, the copper species in the MBC/CuO remained in the Cu (II) state, which may be because the formed Cu (III) was too unstable to be detected [47,48]. For XPS spectra of O 1s (Figure 5c), the peaks located at 530.01, 531.60 and 532.80 eV, which were assigned to the bonding of lattice oxygen (OI) with metal cations, −OH groups (OII), and the adsorbed H_2_O (OIII) on the MBC/CuO surface, respectively [49]. After the degradation reaction, the area of OII and OIII increased with the decrease in OI, implying the surface of MBC/CuO was hydroxylated during the degradation reaction, which was consistent with previous results [13]. The high-resolution XPS spectra of C1s were also investigated in Figure 5d, which could be attributed to C=C, C–C, C–H (284.85 eV); C–OH (285.81 eV); and COOH (288.61 eV), respectively [50]. The peak area of C–OH and COOH decreased in the used MBC/CuO, indicating that C–OH and COOH were converted to CO^▪^ and COO^▪^ using Equations (8) and (9), and became participate during the activation of PS [51].

BC_surface_-OOH + S_2_O_8_^2−^ → BC-OO^•^ + SO_4_^•−^ + HSO_4_^−^ (8)

BC_surface_-OH + S_2_O_8_^2−^ → BC-O^•^ + SO_4_^•−^ + HSO_4_^−^ (9)

**Figure 6:**
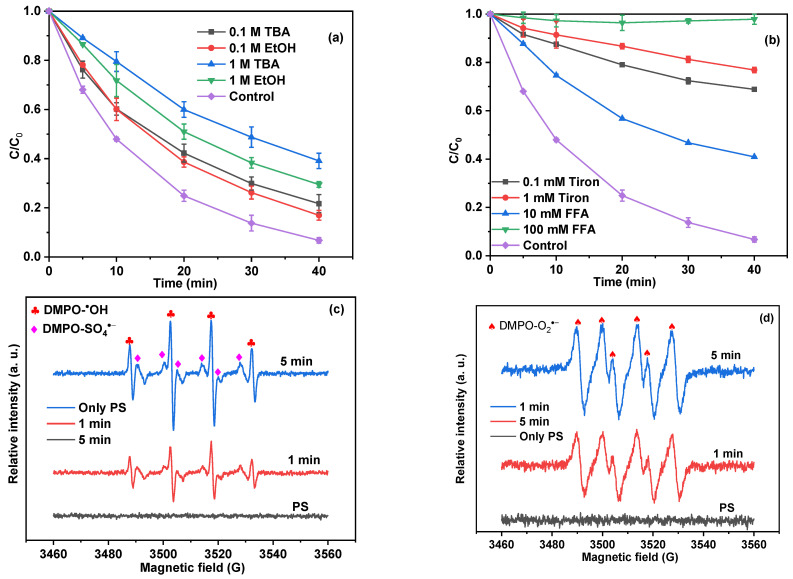

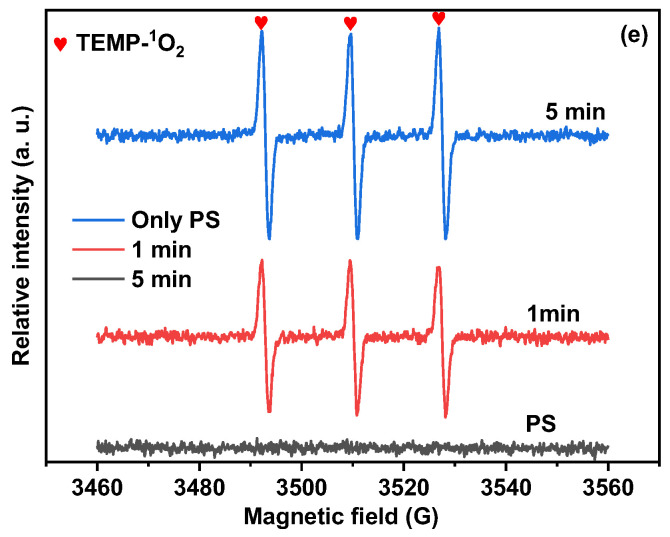
Effects of EtOH and TBA dose on BPA removal (**a**); effects of FFA and tiron dose on BPA removal (**b**); DMPO spin-trapping ESR spectrum of ^•^OH/SO_4_^•−^ (**c**) and ^1^O_2_ (**d**); as well as TEMP spin-trapping ESR spectrum of ^1^O_2_ (**e**). ((MBC/CuO) = 0.1 g/L, (DMPO) = (TEMP) = 100 mM, (PS) = 1 mM, T = 25 °C).

To elucidate the contribution of reactive species on the removal of BPA in the MBC/CuO/PS system, EtOH, TBA, tiron, and FFA were added to the reaction system for radical quenching experiments, and the results are depicted in Figure 6. EtOH could be used as SO_4_^•−^ and ^•^OH scavengers, TBA was often used as a quencher of ^•^OH radical, and tiron was used to quench O_2_^•−^ [52,53], FFA could be used to probe the ^1^O_2_. As depicted in Figure 6a, when different concentrations of EtOH and TBA were introduced, it could be observed that the removal of BPA decreased to different degrees, and the inhibition effect was enhanced with the increase of scavenger dosage. The results indicated that both SO_4_^•−^ and ^•^OH contributed to the degradation pathways in the MBC/CuO/PS system. It should be noted that stronger inhibition was always achieved when TBA was introduced whether at a low concentration (0.1 M) or high concentration (1.0 M), which was inconsistent with most of the reported results [54,55]. This result might be caused by the masking effect of the high viscosity of TBA on the bonding sites dispersed on the catalyst surface [42,56]. As shown in Figure 6b, with the increase in tiron concentration level to 0.1 mM and 1.0 mM, the removal of BPA decreased to 31% and 23%, respectively. In addition, when the FFA concentrations were increased from 10.0 to 100.0 mM, the degradation efficiencies for BPA dropped to 59% and 2%, respectively. These results indicated that a more remarkable inhibition effect of tiron and FFA could be accomplished; thus, O_2_^•−^ and ^1^O_2_ might play a key role in PS the degradation processes.

To further distinguish the radicals and nonradicals generated in the MBC/CuO/PS system, ESR tests were conducted with DMPO and TEMP as the spin-trapping agents to confirm the reactive species. As can be seen from Figure 6c,d, strong signals of DMPO-^•^OH, DMPO-SO_4_^•−^, and DMPO-O_2_^•−^ were observed in the ESR spectra, suggesting the generation of ^•^OH, SO_4_^•−^, and O_2_^•−^ in the MBC/CuO/PS catalytic system. Additionally, obvious TEMP–^1^O_2_ signals were also observed in ESR spectra (Figure 6e), indicating the indispensable roles of ^1^O_2_ nonradicals in the MBC/CuO/PS system. In view of the above results, both free radicals ^•^OH, SO_4_^•−^, and O_2_^•−^ and nonradicals ^1^O_2_ were all involved in the degradation reaction.

Based on the above results, the possible activation mechanism of PS using MBC/CuO was proposed. SO_4_^•−^ were generated through the reaction between PS and Cu^2+^ (Equation (10)) or oxygen-containing functional groups on the BC surface (Equations (8) and (9)), and the generated SO_4_^•−^ would partially react with H_2_O or OH^−^ to generate ^•^OH (Equation (11)) [30]. In addition, S_2_O_8_^2−^ can be hydrolyzed to HSO_5_^−^ via Equation (12) under acidic conditions. Recovery of Cu^2+^ center could be realized by the reaction of Cu^3+^ and HSO_5_^−^ (Equation (13)) [54], which was consistent with the previous XPS results that the copper species in the used catalyst remained in the Cu(II) state. Meanwhile, O_2_^•−^ can be generated by MBC promoting the electron transfer of O_2_ (Equation (14)). Thereafter, ^1^O_2_ was generated by Equations (15) and (16) [11]. As a result, the in situ-generated radicals and nonradicals can work together for the efficient degradation of BPA in MBC/CuO/PS system.

≡Cu^2+^ + S_2_O_8_^2−^ → ≡Cu^3+^ + SO_4_^•−^ + SO_4_^2−^ (10)

SO_4_^•−^ + H_2_O/OH^−^ → SO_4_^2−^ + ^•^OH (11)

S_2_O_8_^2−^ + H_2_O → HSO_5_^−^ + HSO_4_^−^ (12)

≡Cu^3+^ + HSO_5_^−^ → Cu^2+^ + SO_5_^•−^ + H^+^ (13)

BC + O_2_ → O_2_^•−^ + BC^+^ (14)

O_2_^•−^ + O_2_^•−^ + 2H^+^ → ^1^O_2_ + H_2_O_2_ (15)

^•^OH + O_2_^•−^ + 2H^+^ → ^1^O_2_ + OH (16)

### 2.5. Possible Degradation Pathways and Stability

To better understand the degradation pathways of BPA in the present system, UPLC-MS was used to analyze the formed intermediate degradation products of BPA. The main chemical components of the identified degradation intermediates are presented in Figure S4. According to previous studies [57,58,59,60], the possible degradation pathways are shown in Figure S5. Firstly, the formed radicals attacked the aromatic ring and generated a BPA phenoxyl radical, which was further converted to form phenol radicals and P1 through β-scission. Furthermore, hydroxyl groups could be introduced onto the aromatic ring of BPA by adding ^•^OH followed by oxygen mediated H• elimination [60], which could be further oxidized to form P3 with *m*/*z* of 260. Finally, ring opening products were produced, and further partially mineralized into CO_2_ and H_2_O.

Cycle experiments were performed to evaluate the stability of MBC/CuO. After three cycles, the degradation efficiencies of BPA were reduced from 93% (first run) to 71% (second run) and 58% (third run) and (Figure S6a), and *k_obs_* values also decreased from 0.066 to 0.0295 and 0.0208 min^−1^. The reduction of BPA removal may be ascribed to the adsorption of reaction intermediates on the active sites of the catalyst surface during the reaction process, and the oxidation of the functional groups might be another reason for deactivation [61]. In addition, the XRD patterns of the used MBC/CuO could be seen in Figure S6b, and no obvious change could be found after three cycles in comparison with the pristine catalyst, indicating the good stability of MBC/CuO.

### 2.6. Toxicity Evaluation

**Figure 7:**
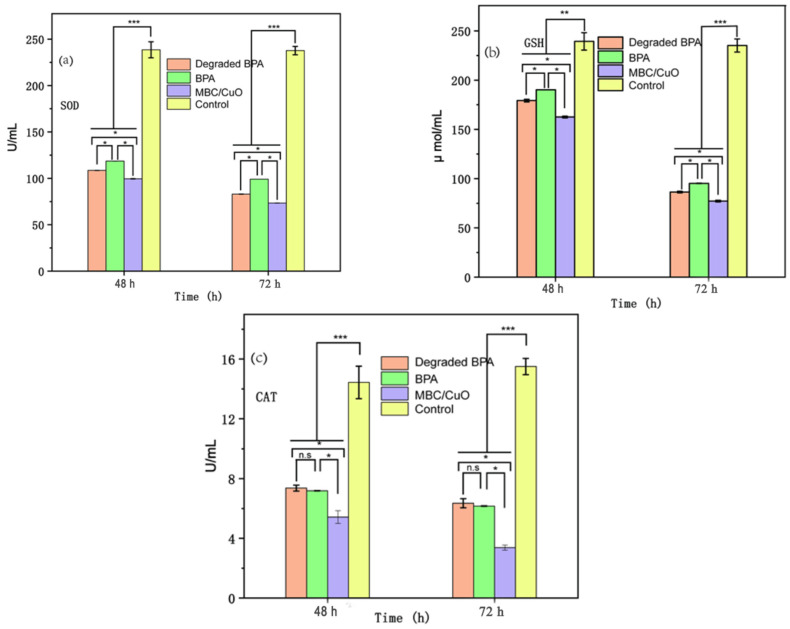
The concentration of total protein homogenate in hemolymph, the enzyme activity of SOD (**a**), GSH (**b**), and CAT (**c**) in control, MBC/CuO, degraded BPA, and BPA treatment groups, respectively, calculated based on the total protein homogenate in hemolymph. Error bars depict the mean ± SD, *n* = 3. n.s. is the abbreviation for no significance, * *p* < 0.05, ** *p* < 0.01, *** *p* < 0.001.

The toxicity evaluation of BPA, MBC/CuO, and its intermediates (degraded BPA) in the reaction system was conducted using 5th instar silkworm larvae. As indicated in Figure S7, the body mass of silkworms was no significant difference in all the four groups including control and treatments, indicating a relatively stable health condition for silkworms during the experiment process. No significant difference was observed between the experimental group and the control group in terms of the body shape of the silkworm (Figure S8). Under healthy conditions, the capacity of cells to produce reactive oxygen species (ROS) and detoxify ROS is balanced. However, under unhealthy conditions, either increased or decreased levels of ROS lead to oxidative stress, which could be harmful because it causes damage to the lipids, proteins, and DNA in the cell. In the present study, this balance was disrupted by BPA. Cellular tissues are harmed by the ROS radicals produced when oxidative stress caused by a toxicant occurs. The antioxidase system is downregulated by these damaging ROS, including GSH, SOD, and CAT levels. SOD is one of the antioxidant enzymes that converts superoxide anions into hydrogen peroxide. SOD was inhibited in cells after BPA exposure as it appeared to be much lower than that was in the control group (Figure 7a). The possible reason for reducing levels of SOD in 48 h exposure was due to the inhibition by excess superoxide anions. As a result, at longer timepoint, the levels of H_2_O_2_ produced by SOD from superoxide anions decreased. As indicated in Figure 7b, in the control group, the levels of GSH were significantly higher than that in the other three contaminants treatments. The activity of GSH decreased constantly and reached the lowest level at 72 h, which are similar to the SOD test. Another essential enzyme for eliminating H_2_O_2_ is GSH [62,63]. It was found that the GSH activity in the control group was higher than that of the BPA treatment group (Figure 7b). According to previous studies [64,65], GSH can be converted to GSSG by GSH–PX in cells, with the GSH–PX concentration rising at the expense of GSH. The concentration of GSH–PX reduced following exposure to BPA, just like CAT (Figure 7c) and SOD. The correlation between the trend of H_2_O_2_ produced by SOD following exposure to BPA and the trend of H_2_O_2_ removed by GSH suggests that the H_2_O_2_ was scavenged by GSH synergistic impact in reducing oxidative damage in organisms [66]. However, the decreased GSH enzyme activity 48 h and 72 h after exposed to three contaminants system suggest that too much H_2_O_2_ may harm the antioxidase (Figure 7c).

**Figure 8:**
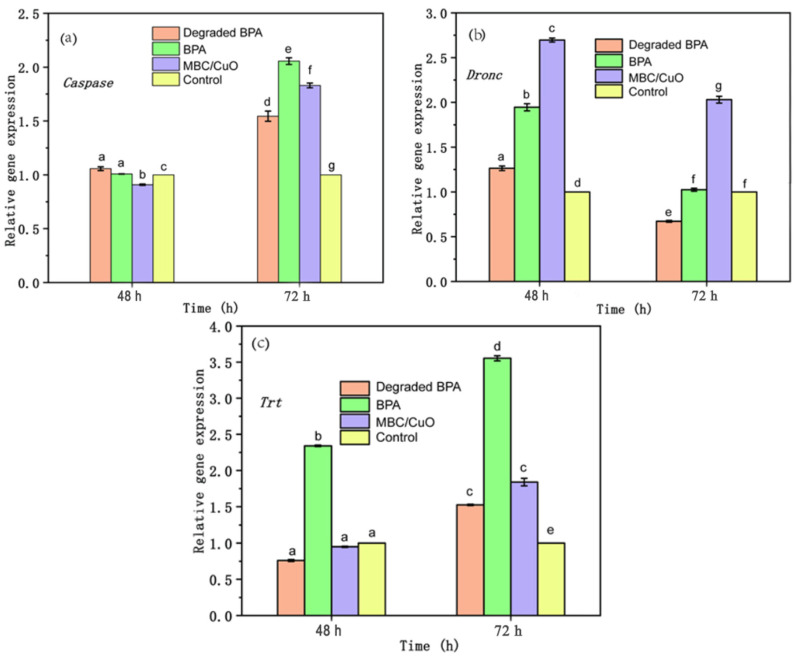
Effects of MBC/CuO, degraded BPA, and BPA, respectively, on the expression of *Caspase*, *Dronc* and *Trt*: The expression level of (**a**) *Caspase*, (**b**) *Dronc*, and (**c**) *Trt* at the indicated developmental stages (48 and 72 h) in treated and control silkworms determined using real-time PCR. Error bars depict the mean ± SD, *n* = 3. Different low case letters above columns indicate statistical differences at *p* < 0.05.

As shown in Figure 8a, the situation is similar to body mass, degraded BPA, and BPA, and MBC/CuO did not change the expression of the *Caspase* gene after 48 h exposure when compared to control. However, the expression of *Caspase* gene was enhanced by the three treatments after 72 h, which suggested that the BPA compound is a potent genotoxicant and triggers cell death. Fortunately, the cell death was reduced when the materials was added in BPA system. It seems that the use of MBC/CuO at considerable levels may have reduce the sensitivity of the genotoxicity with BPA in this study. *Dronc* gene expression was induced by BPA in this study and is consistent with the caspase activity test. The elevation of the *Dronc* gene levels was evidenced in degraded BPA and MBC/CuO treatments as compared with that in control groups after 48 h exposure. The levels of this gene in MBC/CuO treatment were about 2.8 times and 1.5 times higher than that in the control and BPA groups, respectively. Surprisingly, as shown in Figure 8b, BPA induces concentration-dependent *Dronc* gene response, and this effect was completely mitigated by MBC materials in the degraded BPA system, in consistency with the role in the caspase test, although the toxicity of MBC materials alone is still relatively high against nontarget organism silkworms. Another gene (*Trt*) related to apoptosis was also observed in the present study. As indicated in Figure 8c, BPA started to induce the gene expression of *Trt* after 48 h, which was 2.5 times higher than that in control groups. At the 72 h condition, the *Trt* levels were even increased to 3.5 times, but MBC materials inhibited the gene overexpression in the degraded BPA group, which demonstrated a blocking effect. Cell apoptosis is a natural process that allows organisms to self-protect in the event that they become exposed to potentially harmful substances. This innate ability allows organisms to eliminate potentially harmful substances and repair damaged cells. Apoptosis is a process that involves many genes in cells. One of these is the Dronc gene, which is a promoter gene associated with cell apoptosis and is dependent on the caspase pathway. *Dronc* is also known as the key effect in apoptotic cells [67]. The integrated enzyme reactions are triggered when active cys-teinyl aspartate specific proteinases are present. This results in the eventual disintegration of DNA that is located on chromosomes and even cells following exposure of BPA, the expression of the important apoptosis-relevant genes *Dronc* and *Caspase* increased, indicating that these genes were activated by the BPA. This was demonstrated by their overexpression in comparison to the control group. Our findings confirmed this activation (Figure 8c). Telo-merase reverse transcriptase (*Trt*) is more closely associated with growth than it is with apoptosis, and it was also induced by MBC materials. This was reflected by an increased expression in the treatments exposed group and a decreasing expression trend throughout the exposure time. Based on the above results, compared with the original BPA solution, the final BPA solution treated with MBC/CuO/PS system was significantly less toxic to silkworms.

## 3. Materials and Methods

### 3.1. Chemicals and Materials

Na_2_S_2_O_8_, NaOH, NaCl, NaHCO_3_, and sodium humate used as natural organic matter (NOM) were supplied by Sinopharm (Shanghai, China). BPA, methanol (MeOH), ethanol (EtOH), tert-butanol (TBA), furfuryl alcohol (FFA), tiron, and other chemicals were purchased from Sigma-Aldrich (Shanghai, China). Mulberry branches were collected from the mulberry garden of Southwest University. The pH of the solution was measured using a pH meter (FE28-Standard, Mettler Toledo, Switzerland). In addition, ultrapure water was prepared with a Milli-Q ultrapure water system (EMD Millipore, Billerica, MA, USA), and was used for all experiments. A field emission scanning electron microscope (SEM, SU-8010, Hitachi, Japan) was used to observe the surface morphology of the sample. The crystalline phase of the samples was measured using an X-ray powder diffraction (XRD) spectrometer (D/max-2550, Rigaku S2, Japan). The specific surface area and pore structure distribution of MBC and MBC/CuO were obtained from N_2_ adsorption/desorption isotherms (Quantachrome Autosorb-iQ). X-ray photoelectron spectroscopy (XPS) was used to analyze surface chemical states of the elements, which was conducted using an X-ray photoelectron spectrometer (ESCA Lab 250Xi, Thermo Scientific, Oxford, UK).

### 3.2. Preparation of MBC/CuO Catalyst

Firstly, dried mulberry branch powder was heated at 700 °C for 2 h and the heating rate was 10 °C/min in a tube furnace under the protection of N_2_ atmosphere to obtain MBC. Afterward, the mass ratio of CuO to MBC was optimized to be 1:1. Specifically, 3.02 g of Cu(NO_3_)_2_·3H_2_O and 1.0 g of the prepared MBC were dissolved in 50 mL ultrapure water, the precipitation was obtained by dropwise addition of 25 mL of NaOH solution (1 M) and continuous magnetic stirring for 2 h. After standing for 60 min, the solid was collected using suction filtration, and washed repeatedly with ultrapure water and EtOH. The collected solid was then dried and further heated at 200 °C for 2 h under N_2_ atmosphere in the tube furnace.

### 3.3. Degradation Experiment Procedures

The batch experiments were conducted in a 150 mL glass conical flask, which contained 100 mL of mixed solution including MBC/CuO (0.1 g/L), BPA (10 mg/L), and PS (1.0 mM), and the temperature was controlled at 25 °C. For the effect of the initial pH investigation, the pH of the reaction solutions was adjusted to the desired value with 0.1 M NaOH or H_2_SO_4_. At predetermined time intervals, a 1 mL sample was collected and mixed with 0.2 mL MeOH to quench the reaction, and then filtered through a 0.22 µm PTFE membrane before the analysis of the target compound with high-performance liquid chromatography (HPLC). All experiments were performed at least twice.

### 3.4. Toxicity Assessment

A bivoltine strain (DaZao) of B. mori was used in the present study. After hatching, larvae were reared on mulberry leaves at 25–27 °C and 85% relative humidity (H.R.) under a 12 h light/12 h dark photoperiod and harvested at specific developmental stages. On the first day of the 5th instar, 30 healthy and uniform silkworm larvae were randomly selected and divided into 3 groups (10 larvae in each group). They were fed with mulberry leaves sprayed with degraded BPA, BPA, and double deionized water (as a control experiment) until cocooning.

The contents of superoxide dismutase (SOD), catalase (CAT), and glutathione (GSH) were used to evaluate the oxidative stress in silkworm tissues. In present study, levels of enzymes were detected from blood, the location of blood collection from the silkworm can be observed in Figure S1. Relative expression of three apoptosis-related genes (*Dronc*, *Caspase*, and *Trt*) in hemolymph was investigated by quantitative real-time PCR (qRT–PCR).

### 3.5. Analytical Methods

#### 3.5.1. HPLC Analysis of BPA

The concentration of BPA was analyzed on DIONEX UltiMate 3000 HPLC with a C-18 column (Agilent, 5 μm, 250 × 4.6 mm) and equipped with a UV/vis photodiode array detector, and the BPA was detected using UV absorbance at 278 nm. The mobile phase for the detection of BPA consisted of 80% MeOH and 20% water (0.2% HAc) (*v*/*v*) at a constant flow rate of 1.0 mL/min. The column temperature and injection volume were set at 25 °C and 20 μL, respectively.

#### 3.5.2. RNA Isolation and Complementary DNA (cDNA) Synthesis

The RNA samples were obtained using RNAiso Plus (TaKaRa, Dalian, China) in accordance with the manufacturer’s recommendations, and then using DNase I (Invitrogen, Carlsbad, CA, USA) treatment to remove genomic DNA. Using ultraviolet spectrophotometry and gel electrophoresis, the RNA’s integrity and purity were analyzed. An All-in-One™miRNA First-Strand cDNA Synthesis Kit (Gene Copoeia, Rockville, MD, USA) was used to reverse transcribe one microgram of total RNA into cDNA in accordance with the manufacturer’s instructions.

#### 3.5.3. RT–qPCR Analysis

Real-time quantitative PCR (RT–qPCR) was used to determine the target genes’ levels in DaZao. RT–qPCR was performed using the program (95 °C for 3 min, 40 cycles of 95 °C for 10 s, and 60 °C for 30 s, and then a final dissociation) with the cDNA reverse-transcripted from the total RNA of silkworm in the fifth instar larval staged. The RT–qPCR was set up in a 20 μL reaction volume, which included 2 μL template, 10 μL of 2 × NovoStart1 SYBR qPCR SuperMix Plus (Novoprotein), 4 μL of ROX Reference Dye II (ROX II; Novoprotein), 2 μL of specific primers (10 μM), and 2 μL ddH_2_O. Bm-Actin3 was used as the reference gene. Using the cycle threshold value (Ct) and the 2 − ΔCt method, we measured the relative expression level of our target gene, where ΔCt = Ct target gene − CtBmA3. A two-tailed *t*-test was used to compare how these genes’ expressions varied: * *p* ≤ 0.05; ** *p* ≤ 0.01; *** *p* ≤ 0.001. The primers are listed in Table S1 [68]. Using the commercial test kits from the Nanjing Jiancheng Bioengineering Institute, China, the levels of MDA, SOD, CAT, and GSH were measured. The supporting information showed the results for the enzyme activities.

## 4. Conclusions

In this study, waste mulberry branches were successfully converted into BC and used as a support for CuO to synthesize MBC/CuO composites, which were used as PS activators for degradation of BPA. Excellent catalytic activity was observed through MBC/CuO heterogeneous catalyst, 93% of BPA could be removed under optimum conditions within 40 min. Cl^−^ and NOM had a certain degree of inhibitory effect on the degradation of BPA, while HCO_3_^−^ promoted the removal of BPA. The experiment of radical scavenging and ESR demonstrated that both free radicals ^•^OH, SO_4_^•−^ and O_2_^•−^ and nonradicals ^1^O_2_ played an important role in the MBC/CuO reaction system, and the activation mechanism of PS was also elucidated. Furthermore, the toxicity of BPA, MBC/CuO, and the final BPA degradation solution against the silkworm larvae were studied. It was found that the toxicity of BPA treated by the MBC/CuO/PS system was lower than that of the original BPA solution, and the synthesized MBC/CuO composite was also low-toxic and environmentally friendly. These findings helped clarify the viability of recycling waste mulberry branches into environmentally friendly functional materials and the potential environmental risks.

## Data Availability

The data that support the findings of this study are available within the article and the Supplementary Information. Further data are available from the corresponding author upon reasonable request.

## Supplementary Materials

The following supporting information can be downloaded at: https://www.mdpi.com/article/10.3390/ijms24043609/s1.
