# Supplementary material for: Synthesis of an Environmentally Friendly Modified Mulberry Branch-Derived Biochar Composite: High Degradation Efficiency of BPA and Mitigation of Toxicity in Silkworm Larvae
Source: Int J Mol Sci. 2023 Feb 10;24(4):3609. doi: 10.3390/ijms24043609 (PMC9961375; doi:10.3390/ijms24043609)
Supplement: Supplementary file 1 [file ijms-24-03609-s001.zip › ijms-2168982-supplementary.pdf]

Supplementary information

for

Synthesis of an Environmentally Friendly Modified Mulberry Branch-Derived Biochar Composite: High Degradation Efficiency of BPA and Mitigation of Toxicity in Silkworm Larvae

Han Qu[1,4], Lin Chen[1], Fujian Yang[1], Jiangwei Zhu[2], Chengdu Qi[3,*], Guilong Peng[1,*]

[1]State Key Laboratory of Silkworm Genome Biology, Key Laboratory of Sericultural Biology and Genetic Breeding, Ministry of Agriculture and Rural Affairs, College of Sericulture, Textile and Biomass Sciences, Southwest University, Chongqing, 400715, China

[2]Co-Innovation Center for Sustainable Forestry in Southern China, Nanjing Forestry University, Nanjing 210037, China

[3]School of Environment, Nanjing Normal University, Nanjing, 210023, China

[4]Key Laboratory of Eco-Environment of Three Gorges Region of Ministry of Education, Chongqing University, Chongqing, 400045, China

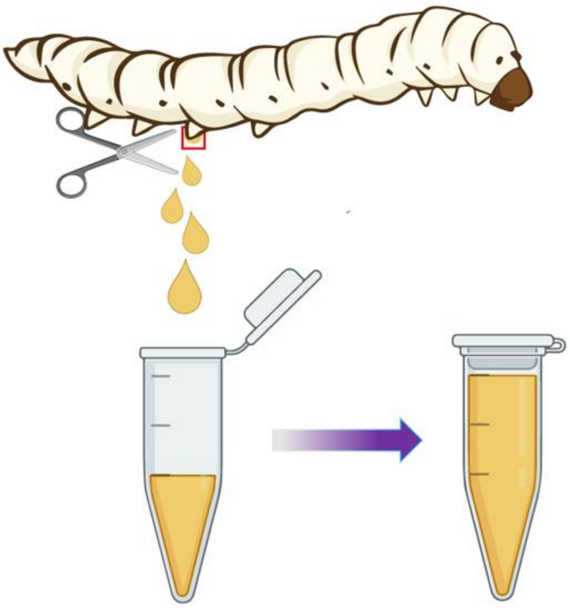

Figure S1. Schematic figure showing where the blood was collected from the silkworm's body, the samples of blood were collected at 48 h and 72 h.

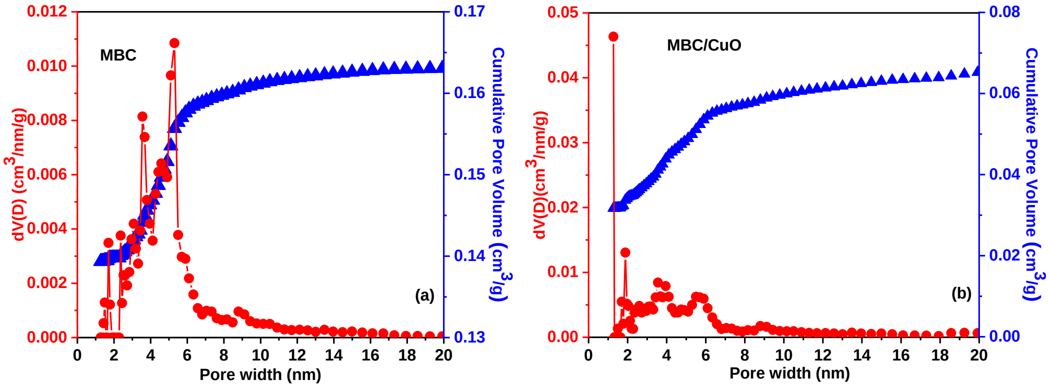

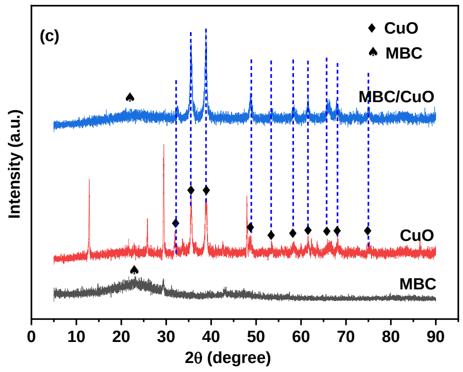

Figure S2. BJH pore size distribution of MBC (a) and MBC/CuO (b), as well as XRD patterns of MBC, CuO and MBC/CuO (c).

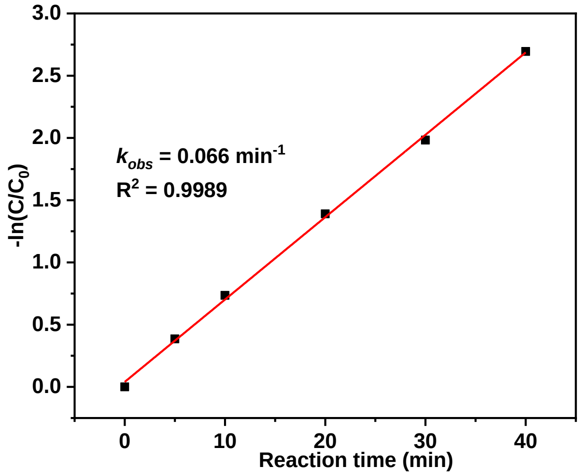

Figure S3. The pseudo-first-order rate of BPA degradation by MBC/CuO/PS system.

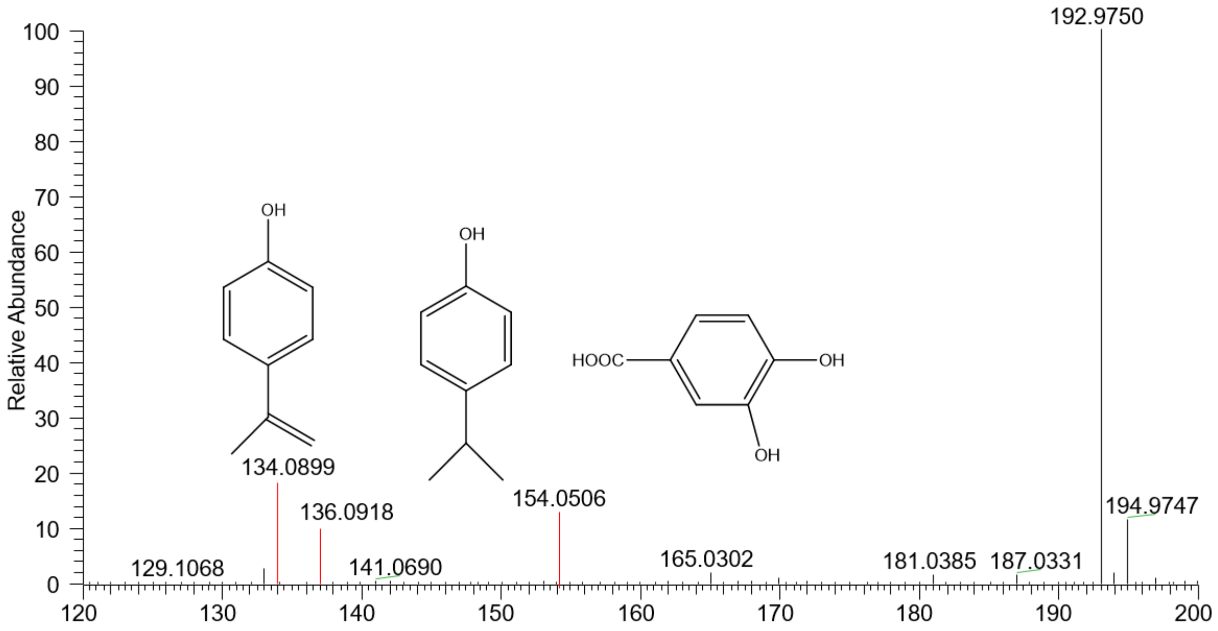

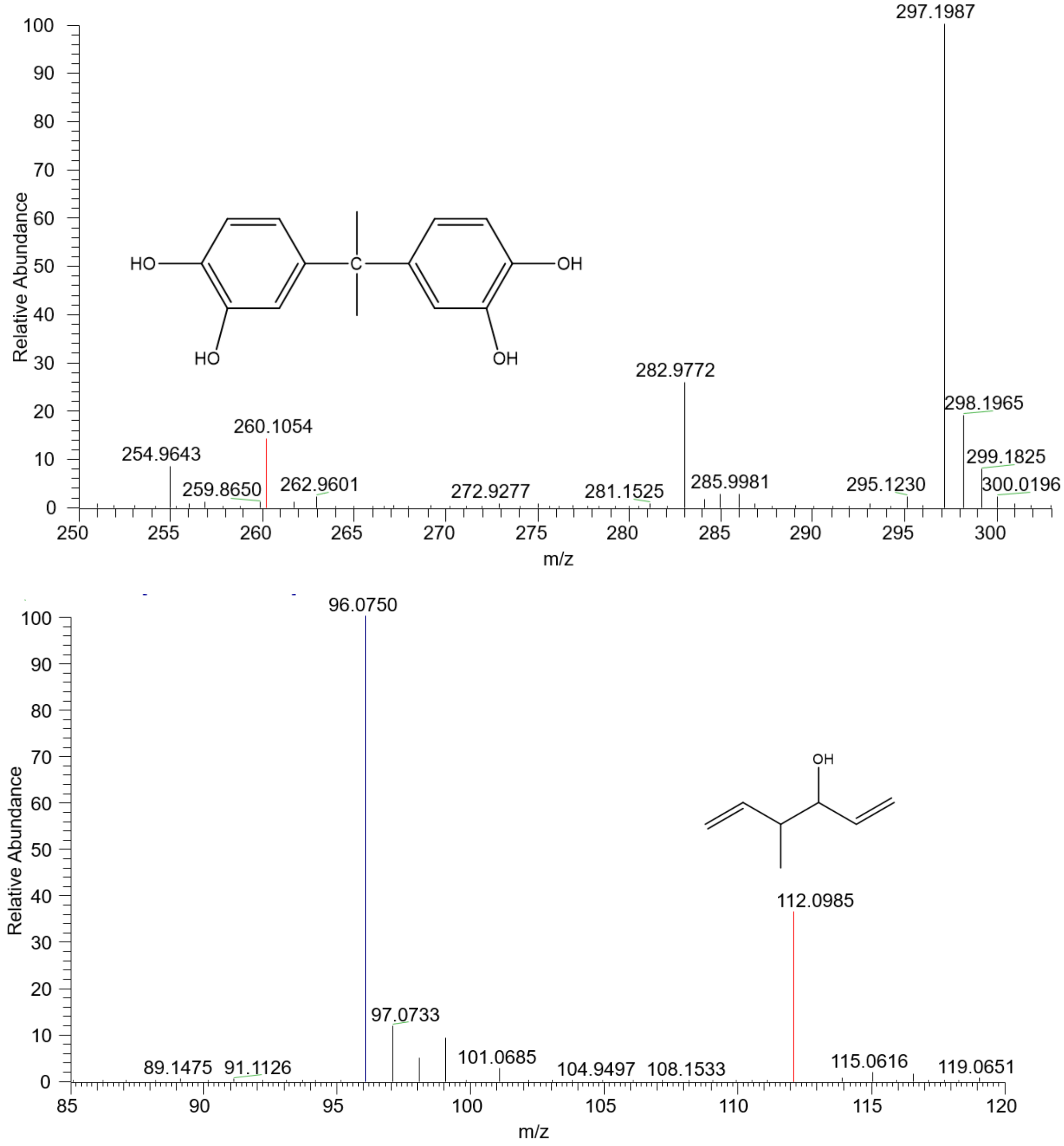

Figure S4. ESI mass spectra of various degraded products as determined by UPLC–MS.

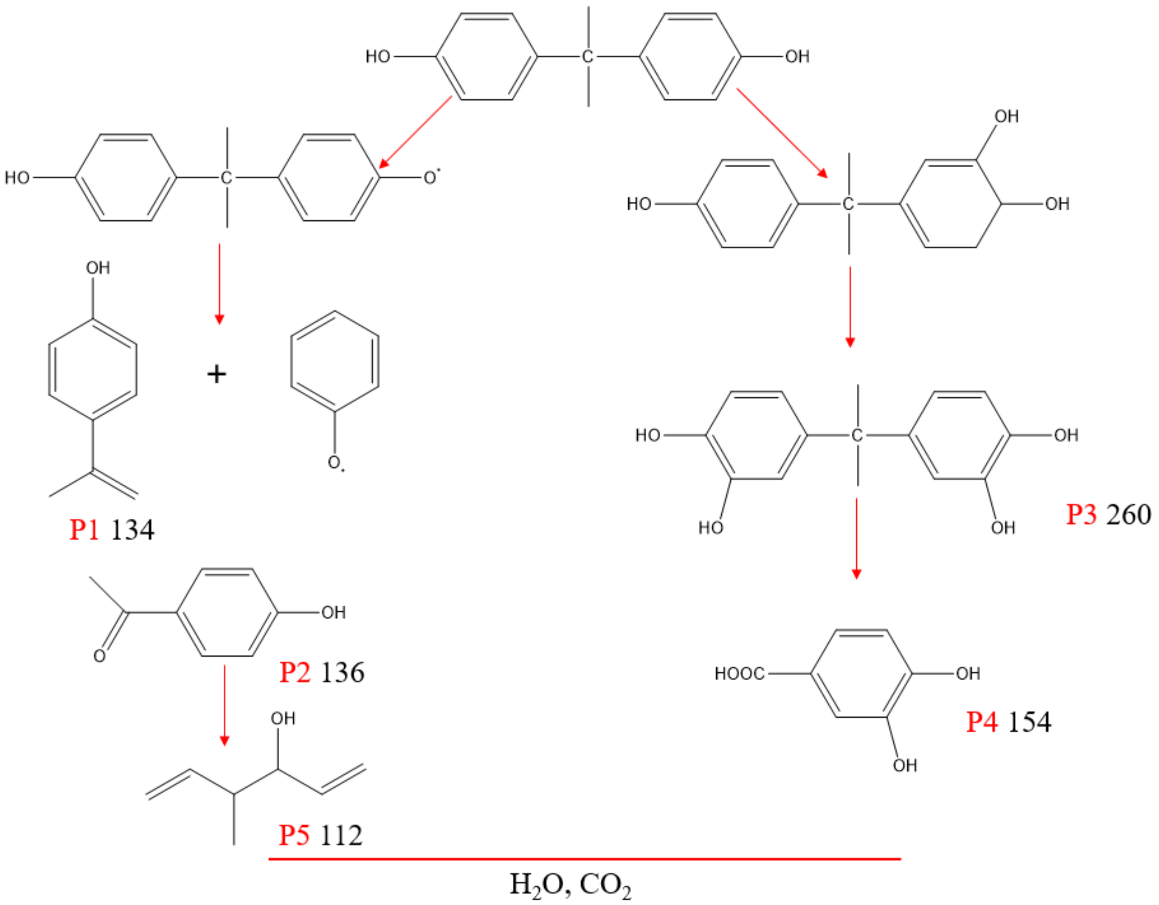

Figure S5. Possible BPA degradation pathways by in the MBC/CuO/PS system.

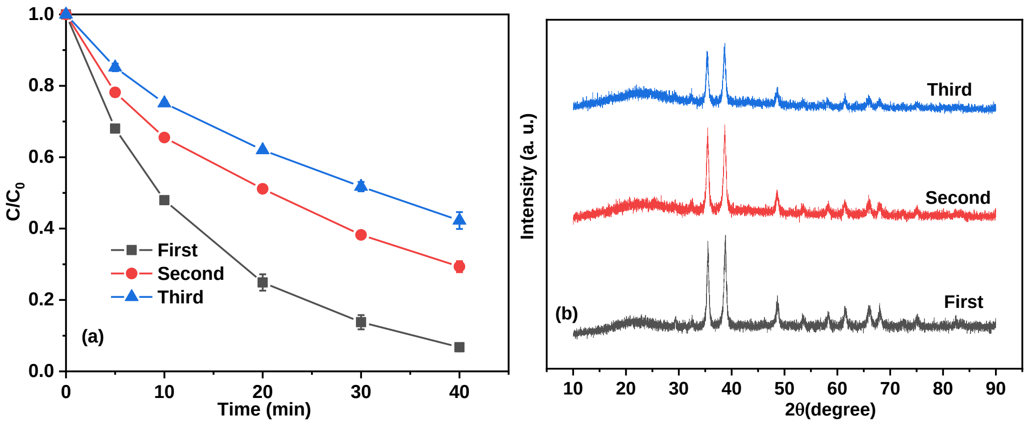

Figure S6. The reusability of MBC/CuO (a) and XRD patterns of reused MBC/CuO (b).

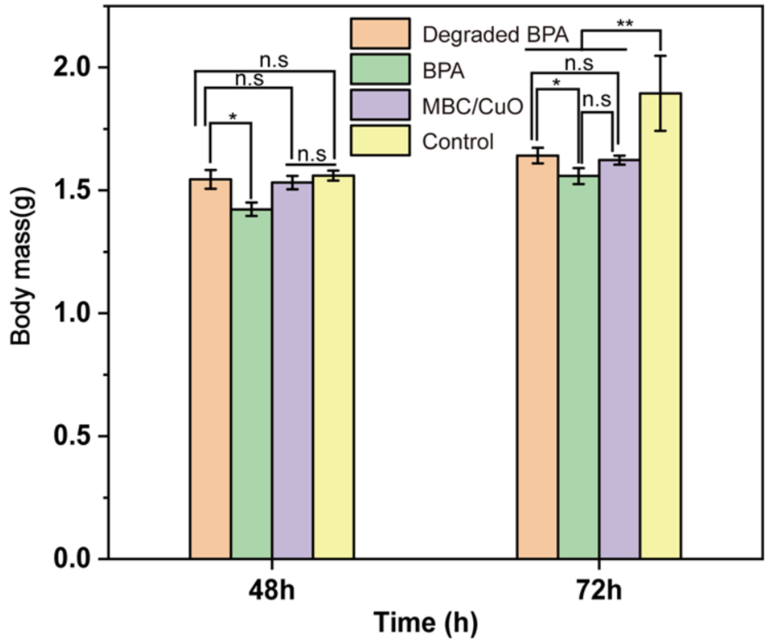

Figure S7. Body mass of the silkworms.

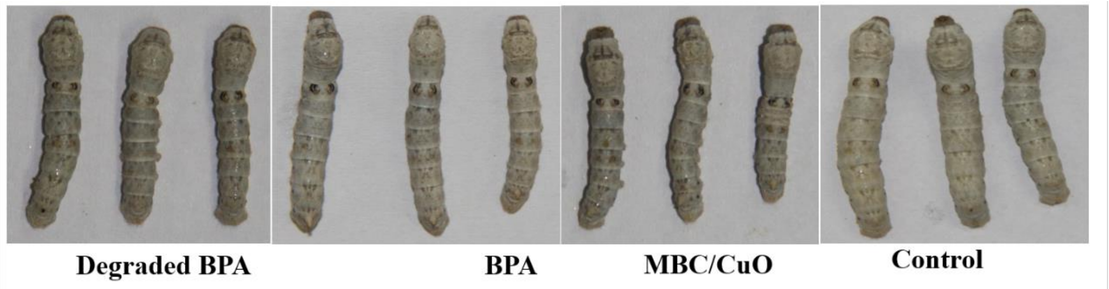

Figure S8. Pictures of silkworm larvae after the intake of degraded BPA, BPA , MBC/CuO, and water (control) for 96 h.

Table S1. qRT- PCR Primer Pairs.

Gene name	Sense primer (5’-3’)	Antisense primer (5’-3’)	Primer size(bp)
Caspase	CTGAGGCCGTGTGCAATTAT	CCTTCGTCTTCATTGCCGTT	124
Trt	ACATCTCCATTGCTGCTTAT	GGGTCACCGTCAGAATCAC	247
Dronc	CCTCGTGAACGGAAACGAAT	GGGCTGTTTGGATCAAGGTC	131
Bm-Actin3	CTTGGGTATGGAAGCCAACG	TGGTACCACCGGACAATACG	113

Table S2. Specific surface areas and pore characteristics of samples.

Sample	BET Surface Area (m^2/g)	Pore volume (cm^3/g)	Average pore size (nm)
BC	84.9	0.17	2.4
BC/CuO	82.9	0.079	3.8

Table S3. A comparison of BPA degradation between BC/CuO/PS system in this study and other systems reported in previous studies.

BPA concentrations	Catalysts concentrations	Oxidants concentrations	k_{obs} (min^{-1})	Removal efficiency (%)	Reaction time (min)	Ref.
0.1 mmol/L	$CuFeO_2$ (1.0 g/L)	H_2O_2(20 mmol/L)	0.047	99.2	120	[69]
20 mg/L	$Fe_{0.8}Co_{2.2}O_4$ (0.1 g/L)	PMS(0.2 g/L)	0.049	95	60	[70]
0.1 mmol/L	BC (0.1 g/L)	PMS (1.0 g/L)	0.022	46.26	60	[26]
0.1 mmol/L	BC/CuO (0.4g /L)	PS (4 mmol/L)	0.607	100	180	[71]
0.1 mmol/L	Fe_3O_4/BC (0.4 g/L)	PS (4.0 mmol/L)	0.0037	33.91	180	[71]
10 mg/L	BC/CuO (0.1 g/L)	PS (2.0 mmol/L)	0.066	93	40	This study

Table S4 The concentrations of the dissolved Cu^{2+} at different initial pH.

Initial pH	2.85	6.23	10.46
Leaching Cu^{2+} (mg/L)	7.85	2.12	0.06

Table S5. The change of pH during the reaction.

Before reaction	After reaction
2.85	4.11
6.23	5.48
10.46	6.53

Table S6. Physical-chemical parameters of different waters used in this study.

Parameters	Ultrapure water	River water	Lake water	Tap water
pH	6.86	7.26	8.11	7.81
TOC (mg L^{-1})	n.d.	15.26	28.63	8.12
Cl^- (mg L^{-1})	1.70	116.35	175.61	84.16
SO_4^{2-} (mg L^{-1})	n.d.	16.32	72.06	35.08
NO_3^- (mg L^{-1})	n.d.	0.77	8.45	2.69
HCO_3^- (mg L^{-1})	n.d.	156.91	205.36	26.72
PO_4^{3-} (mg L^{-1})	n.d.	1.12	2.33	n.d.

n.d.: Not detected.

69. X. Zhang, Y. Ding, H. Tang, X. Han, L. Zhu, N. Wang, Degradation of bisphenol A by hydrogen peroxide activated with CuFeO2 microparticles as a heterogeneous Fenton-like catalyst: Efficiency, stability and mechanism, Chemical Engineering Journal, 2014, 236, 251-262.
70 L. He, Z. Liu, J. Hu, C. Qin, L. Yao, Y. Zhang, Y. Piao, Sugarcane biochar as novel catalyst for highly efficient oxidative removal of organic compounds in water, Chemical Engineering Journal, 2021, 405, 126895–126908.
71. H. Luo, Q. Lin, X. Zhang, Z. Huang, H. Fu, R. Xiao, S.-S. Liu, Determining the key factors of nonradical pathway in activation of persulfate by metal-biochar nanocomposites for bisphenol A degradation, Chemical Engineering Journal, 2020, 391, 123555.
